# Traumatic Stress-Induced Vulnerability to Addiction: Critical Role of the Dynorphin/Kappa Opioid Receptor System

**DOI:** 10.3389/fphar.2022.856672

**Published:** 2022-04-27

**Authors:** Claire Leconte, Raymond Mongeau, Florence Noble

**Affiliations:** Université Paris Cité, INSERM, CNRS, T3S, Paris, France

**Keywords:** addiction, PTSD–posttraumatic stress disorder, traumatic stress disorder, kappa opiate receptor, dynorphin

## Abstract

Substance use disorders (SUD) may emerge from an individual’s attempt to limit negative affective states and symptoms linked to stress. Indeed, SUD is highly comorbid with chronic stress, traumatic stress, or post-traumatic stress disorder (PTSD), and treatments approved for each pathology individually often failed to have a therapeutic efficiency in such comorbid patients. The kappa-opioid receptor (KOR) and its endogenous ligand dynorphin (DYN), seem to play a key role in the occurrence of this comorbidity. The DYN/KOR function is increased either in traumatic stress or during drug use, dependence acquisition and DYN is released during stress. The behavioural effects of stress related to the DYN/KOR system include anxiety, dissociative and depressive symptoms, as well as increased conditioned fear response. Furthermore, the DYN/KOR system is implicated in negative reinforcement after the euphoric effects of a drug of abuse ends. During chronic drug consumption DYN/KOR functions increase and facilitate tolerance and dependence. The drug-seeking behaviour induced by KOR activation can be retrieved either during the development of an addictive behaviour, or during relapse after withdrawal. DYN is known to be one of the most powerful negative modulators of dopamine signalling, notably in brain structures implicated in both reward and fear circuitries. KOR are also acting as inhibitory heteroreceptors on serotonin neurons. Moreover, the DYN/KOR system cross-regulate with corticotropin-releasing factor in the brain. The sexual dimorphism of the DYN/KOR system could be the cause of the gender differences observed in patients with SUD or/and traumatic stress-related pathologies. This review underlies experimental and clinical results emphasizing the DYN/KOR system as common mechanisms shared by SUD or/and traumatic stress-related pathologies, and suggests KOR antagonist as a new pharmacological strategy to treat this comorbidity.

## Introduction

Post-traumatic stress disorder (PTSD) and substance use disorders (SUD) are frequently comorbid (María-Ríos and Morrow, 2020; Hien et al., 2021). Indeed, in a civilian population study, SUD lifetime prevalence ranges from 25% to 43% in persons with PTSD, compared with 8%–25% in the general population (Jacobsen et al., 2001). This comorbidity is often associated with more severe clinical profiles compared with either diagnosis alone. Among PTSD patients, the most common SUD is alcoholism: from 24% of patients in the general population (Mills et al., 2006) to 75% in combat veterans (Jacobsen et al., 2001), however, cocaine and heroin use disorders are also highly prevalent (Dworkin et al., 2018). Moreover, 33% of individuals with an opioid use disorder have experienced PTSD (Mills et al., 2006), with 92% of heroin dependent patients exposed to traumatic stress (Mills et al., 2018). In the last decade, the co-occurrence of PTSD and SUD has been well documented, although only few studies have investigated the shared mechanisms. Persons with SUD are all predisposed to traumatic events exposure (Cottler et al., 1992), and inversely, PTSD induces vulnerability to SUD (María-Ríos and Morrow, 2020). At the clinical level, several situations are encountered: PTSD may precede SUD, or inversely, SUD may precede PTSD (Brady et al., 1998), and when PTSD precedes cocaine use for example, PTSD symptoms are often more severe (Brady et al., 1998). The relationship between PTSD and SUD can be explained in part by the self-medication hypothesis: most patients report that the use of drugs, such as alcohol or opiates, can reduce stress symptoms (e.g., insomnia, tachycardia, uncontrolled trembling, hypervigilance…) (Khantzian, 1985, 1997; Volpicelli et al., 1999; Danovitch, 2016).

The fact that there are so many neurobiological and biochemical factors implicated in the development of PTSD and SUD comorbidity makes the treatment of such comorbid situation difficult to codify. Although exposure therapy (*i.e*., exposure to trauma-related stimuli inducing an effective extinction of fear memories) is a highly effective treatment for PTSD alone (Cusack et al., 2016), it appears to be less effective in the SUD/PTSD comorbidity (Simpson et al., 2017). Integrated cognitive behavioural therapy of SUD/PTSD patients began to develop (McGovern et al., 2011, 2015; Roberts et al., 2016), unfortunately, it had no effect on PTSD symptoms. Concerning pharmacological treatments, diverse medications were tested with negative results in alcohol use disorders associated with PTSD (Taylor et al., 2017), with the exception of two molecules. First, sertraline treatment (a selective serotonin reuptake inhibitor; SSRI) combined with cognitive-behavioural therapy reduces both PTSD and alcohol use disorder severity (Hien et al., 2015). Second, naltrexone in combination with prolonged exposure therapy for PTSD demonstrates a beneficial effect 6 months later, for alcohol drinking outcomes (Foa et al., 2013). Naltrexone, as naloxone, is a non-selective mu (µ, MOR), delta (δ, DOR), and kappa (κ, KOR) opioid receptor antagonist, approved for the treatment of alcohol and opioid use disorders (Sudakin, 2016), and is actually tested (in co-treatment with buprenorphine), in a phase 2 clinical trial, to treat alcohol use disorder comorbid with PTSD (NCT03852628, 2019). Interestingly, the pharmacological industry was prompted to develop KOR antagonists to treat stress-induced relapse of cocaine, alcohol, and tobacco (Carroll and Carlezon, 2013; Banks, 2020). To our knowledge, a pharmacological strategy aiming selective KOR antagonism has not yet been explored to treat SUD/PTSD comorbidity. One goal of the present review is to stimulate research in this direction.

Although the mechanisms underlying PTSD/SUD association has been extensively reviewed before (Jacobsen et al., 2001; María-Ríos and Morrow, 2020), understanding better how traumatic stress and more generally PTSD can predispose to SUD would allow to design more effective treatment strategies aimed specifically at patients vulnerable to comorbid psychiatric disorders. In this review, we thus focus on both preclinical and clinical research related to the modulation of the KOR system, and its endogenous ligand dynorphin (DYN), in relation with stress and addiction. The interest for this topic has been growing recently (Helal et al., 2017; Karkhanis et al., 2017; Jacobson et al., 2018; Lanius et al., 2018; Valentino and Volkow, 2018; Beck et al., 2019; Margolis and Karkhanis, 2019; Tejeda and Bonci, 2019; Anderson, 2020; Escobar et al., 2020; Nagase and Saitoh, 2020). We will thus list here some of the most relevant literature on the DYN/KOR system in pain, dysphoria and psychiatric disorders. We will pay a special interest to gender differences. It is necessary to examine in detail the involvement of DYN/KOR in stress-related behaviours, on the brain circuits mediating fear and the differential effects of KOR agonists vs antagonists in addiction. The involvement of KOR in traumatic stress-induced drug reinstatement is also particularly interesting.

## The Dynorphin/Kappa-Opioid Receptor System

Beside the hedonic state that often leads to addictive behaviours, the opioid system is involved in a wide range of physiological functions. Opioids, mainly non-selective agonists with an important MOR affinity, induce analgesia, sedation, respiratory depression, bradycardia, nausea, vomiting, and reduction in gastric motility (Valentino and Volkow, 2018). Opioid receptors belong to G-coupled receptors family with the MOR, DOR, and the KOR subtypes (Valentino and Volkow, 2018), to which we can add the nociceptin opioid receptor (NOR) subtype (Pathan and Williams, 2012). Exogenous ligands with agonist and/or antagonist properties, that are more or less selective, have been largely investigated (including morphine and heroin as MOR agonists). Each receptor possesses its own selective endogenous ligands: endorphin for MOR (with low affinities to DOR and KOR), enkephalins with a higher DOR affinity, and the nociceptin/orphanin FQ for NOR. DYN is derived from pro-dynorphin (PDYN) and is the only endogenous ligand with a high affinity for KOR. Among specific KOR agonists used in preclinical research (Banks, 2020), we can cite salvinorin A, the active principle of *Salvia divinorum* (Roach and Shenvi, 2018) and the synthetic analogue U50,488 (Karkhanis et al., 2017).

**FIGURE 1:**
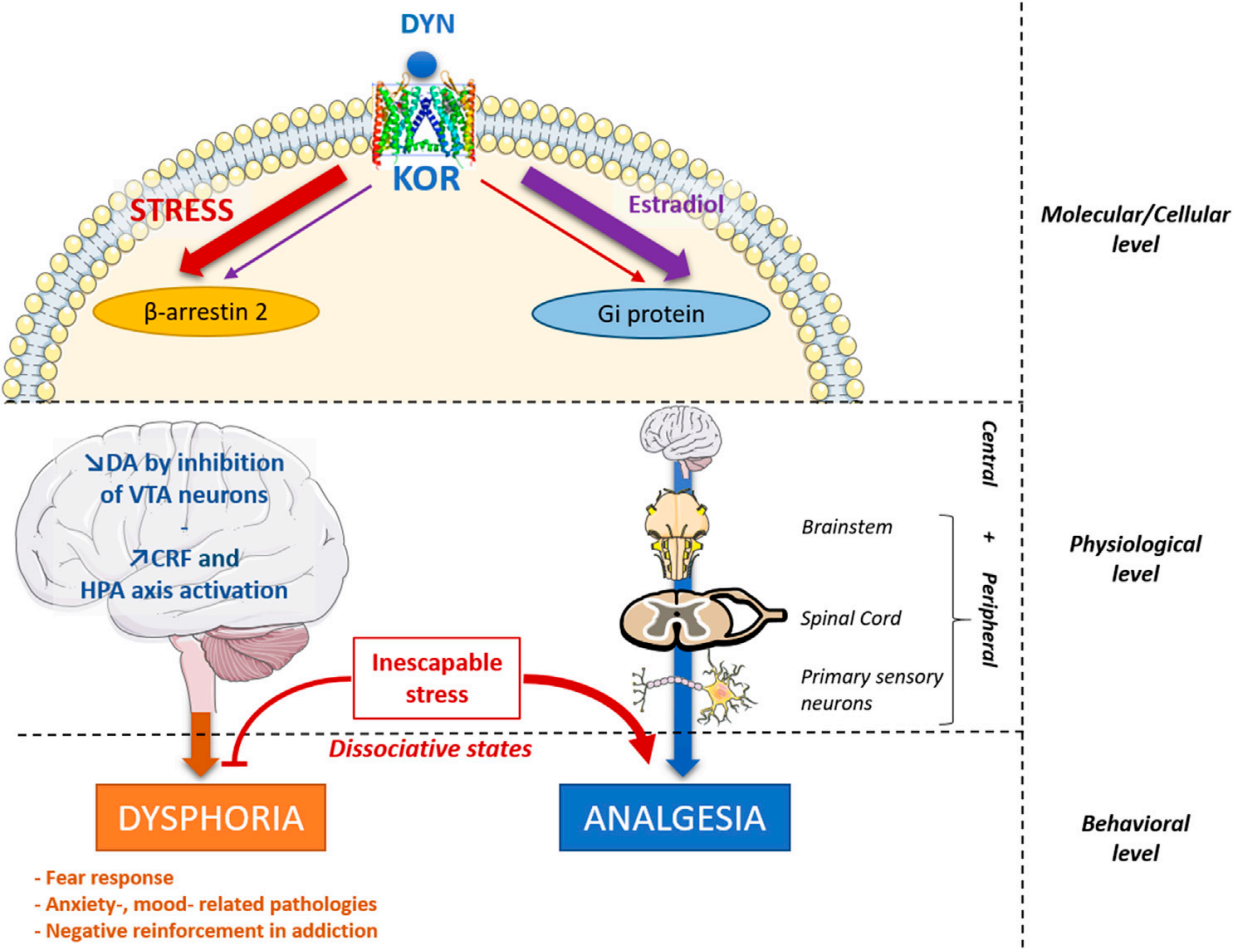
Proposed integrative view of DYN/KOR system main effects and regulations. Dynorphin (DYN) is mainly released by stress and binds to KOR, which triggers its effects through two signaling pathways: the β-arrestin-2 pathway and the Gi protein signaling pathway. Some compounds can create an imbalance between these two main effects, such as estradiol at the molecular level that decrease β-arrestin-2 signaling and increase Gi protein signaling (Abraham A. D. et al., 2018), promoting analgesic effects and decreasing dysphoric effects. Similarly, G-protein biased KOR agonists tend to promote analgesia without dysphoric side effects (Brust et al., 2016; Spetea et al., 2017) KOR also mediate central and peripheral analgesia, by acting on primary sensory neurons, the spinal cord and the brainstem. In turn, dysphoric effects are potentially induced through a DA release decrease in the brain by inhibiting the VTA dopaminergic neurons and by an increased release of CRF and HPA axis activation. Stress-induced analgesia is KOR dependent in the case of inescapable stress. This may somehow be related to dissociative states, following an emotional shutdown, thought to involve KOR activation, increasing opioid dependent analgesia and producing alterations of mood and perception (Lanius et al., 2018). CRF, Corticotropin Releasing Factor; DA, dopamine; HPA axis, Hypothalamo-Pituitary-Adrenal axis; KOR, Kappa Opioid Receptor; VTA, ventral Tegmental Area.

The DYN/KOR system has a wide distribution in central and peripheral nervous systems (Chen et al., 2020) and is implicated in numerous physiological functions: *e.g.* pain perception, dysphoria, neuroendocrine regulation, extrapyramidal motor control, cardiovascular function, respiration, water balance system (diuresis), temperature regulation and feeding behaviour (Fallon and Leslie, 1986). Recently, it has also been shown that KOR activation could decrease the differentiation process during neurogenesis (Xu et al., 2021), and that dynorphin promotes both developmental and stress-induced oligodendrocyte precursor cell differentiation and myelination in the striatum (Osso et al., 2021). As other opioid receptors, KOR mediate peripheral analgesia, by acting on the pain pathway at primary sensory neurons, the spinal cord and the brainstem (Figure 1). KOR mediate also central analgesia, at the amygdala, the parietal cortex and the rostral ventromedial medulla (Cahill et al., 2014). Although pain treatment using KOR agonists seems advantageous compared to the clinically used MOR agonists (no addiction, no respiratory depression), these agonists are problematic in clinic because of their adverse effects on mood and sedation. However, some promising mixed KOR/DOR agonists (Atigari et al., 2021) or KOR biased-agonists are currently being developed (Valentino and Volkow, 2018).

Opioid receptors are coupled to several signalling pathways, including Gi/o-protein-dependent signalling and β-arrestin-2-related signalling (Figure 1). Briefly, following an agonist activation, KOR promotes the Gα subunit and Gβγ subunits dissociation. The Gα subunit interact with various intracellular downstream effectors, as adenylyl cyclases and cGMP phosphodiesterase and possess a GTPase intrinsic activity. Gβγ subunits interact with GRKs, G-protein-coupled phosphoinosite 3 kinase (PI3K) and mitogen-activated protein kinases (MAPK). In addition, the β-arrestin 2 regulates KOR signalling through desensitization and internalization, recruiting also MAPK pathways as p38 stress kinase and extracellular signal-regulated kinases 1 and 2 (ERK 1/2) (Bruchas and Chavkin, 2010; Faouzi et al., 2020).

Interestingly, differences in KOR trafficking have been noted for different agonists: dynorphins, U50,488, salvinorin A, TRK-820, and 3FLB (Jordan et al., 2000; Wang et al., 2005; Chen et al., 2007). It has been proposed that Gi signalling mediates KOR-induced analgesia, while the β-arrestin 2-related signalling mediates dysphoria (Bruchas and Chavkin, 2010). As a matter of fact, the dysphoric effects of KOR activation required arrestin-dependent p38α MAPK activation in ventral tegmental area (VTA) dopaminergic neurons (Ehrich et al., 2015). On the other hand, some partial KOR agonists, promoting Gi-protein signalling, are able to induce antinociception without inducing significant sedative or dysphoric effects (Brust et al., 2016; Spetea et al., 2017). These studies, suggests that distinct signalling pathways inducing differential behavioural and/or physiological effects could be taken into account to create biased-agonists which would improve analgesia while reducing the risk of adverse effects. Similarly, MOR biased agonists have also been investigated (Faouzi et al., 2020). In 1999, it has been demonstrated an improved antinociceptive effect of morphine in β-arrestin2 KO mice (Bohn et al., 1999). However, although MOR G-protein biased agonist were design in order to enhance and/or to prolong antinociception and to decrease tolerance, some recent studies point out controversial results for respiratory depression, constipation or withdrawal (Hill et al., 2018; Kliewer et al., 2019, 2020). As a matter of fact, G-biased KOR agonists lacking addictive properties and dysphoric effects could be much more promising drugs to treat pain (Bruchas and Chavkin, 2010; Valentino and Volkow, 2018; Faouzi et al., 2020).

Apart from the above effects, the DYN/KOR system is involved in several psychiatric diseases, including major depressive disorder (Callaghan et al., 2018; Jacobson et al., 2018), epilepsy (André et al., 2018), schizophrenia (Clark and Abi-Dargham, 2019), borderline personality disorder (Anderson, 2020) and addiction (Anderson and Becker, 2017; Norman and D’Souza, 2017; Victorri-Vigneau et al., 2018). It is also involved in inflammatory diseases (Beck et al., 2019; Coffeen and Pellicer, 2019). Indeed, it is known that the DYN/KOR system is implicated in oxidative stress enzyme activities (Dang et al., 2018), and in the activation of the hypothalamo-pituitary-adrenal (HPA) axis that modulate inflammation [Figure 1; (Fuller and Leander, 1984; Song and Takemori, 1992; Bruchas et al., 2009]. Nevertheless, DYN may also act as an anti-inflammatory compound through the potentiation of glucocorticoids action, and by promoting brain microglial polarization toward an anti-inflammatory M2 phenotype (Liu et al., 2020).

Consistently, DYN is released during prolonged or intense stress (Figure 1) and induces anxiety-like or depression-like behaviours (Van’t Veer and Carlezon, 2013). Interestingly, because of its dysphoric and aversive effects, the KOR system was argued to contribute to the negative affective states induced by pain, driving mood-related pathologies associated with chronic pain (Cahill et al., 2014). Furthermore, in humans, KOR agonists could produce a dissociative-like syndrome, a state occurring during an inescapable traumatic experience (Lanius et al., 2018). These dysphoric effects are thought to be the consequence of a dopamine (DA) depletion in the reward and the fear circuits (Karkhanis et al., 2017; Lanius et al., 2018; Escobar et al., 2020). KOR have been found to be expressed in DA neurons of the nucleus accumbens (NAc), the ventral tegmental area (VTA), the caudate putamen and the substantia nigra (Chen et al., 2020). Indeed, DYN is known to be one of the most powerful negative modulators of DA signalling. It triggers a hyperpolarization in VTA dopaminergic neurons, decreasing DA release in the NAc, the basolateral amygdala (BLA), the medial prefrontal cortex (mPFC), brain structures implicated in both fear and reward (Karkhanis et al., 2017; Lanius et al., 2018; Escobar et al., 2020).

In contrast to either MOR or DOR agonists that induce a hedonic state and participate in positive reinforcement, KOR agonists are generally aversive: their activation leads to anti-reward effects that trigger negative reinforcement (Fields and Margolis, 2015; Karkhanis et al., 2017). Furthermore, the DYN/KOR function is increased during chronic stress or drug dependence development and leads to drug-seeking (Carroll and Carlezon, 2013; Karkhanis et al., 2017). This is why some KOR antagonists seem promising pharmacological strategies in clinic, to reduce the risk of stress-induced relapse during alcohol and cocaine withdrawal, smoking as well as for gambling cessation (Carroll and Carlezon, 2013; Anderson and Becker, 2017; Norman and D’Souza, 2017; Victorri-Vigneau et al., 2018; Banks, 2020; Krystal et al., 2020).

Differences in signalling properties and between selective KOR antagonists were observed. Prototypical potent and selective KOR antagonists such as naltrexone-related antagonists [norbinaltorphimine (nor-BNI), 5′-guanidinonaltrindole (GNTI), …], or (3R,4R)-dimethyl-4-(3-hydroxyphenyl) piperidine-based (JDTic), were used in preclinical studies to dissect properties of the DYN/KOR system. They possess slow onset and long duration of action that require c-Jun N-terminal Kinase (JNK) activation (Bruchas and Chavkin, 2010). They also possess low brain penetration and undesirable side effect, that made them inadequate for clinical trials (Carroll and Carlezon, 2013; Jacobson et al., 2020). CERC-501, used in phase 1 clinical trials, does not share nor-BNI or JDTic pharmacological properties (Carroll and Carlezon, 2013; Jacobson et al., 2020). Nevertheless, this last compound, a KOR antagonist that have low affinities for MOR and DOR (Banks, 2020), failed to attenuate cocaine craving (Reed et al., 2018), or cigarette smoking and craving (Jones et al., 2020).

Another issue, complicating the therapeutic use of KOR ligands, concerns gender differences. KOR triggers a sex-dependent response for both analgesia and dysphoria. The first clinical trials on KOR agonists as analgesics were done on men, consequently, women specific responses to KOR agonists were largely unknown until years 2000s. Indeed, KOR agonists have low or inconsistent effects on pain in women and female rodents although its analgesic efficiency could be related to the oestrous cycle or the sex-dependant MOR/KOR heterodimerization (Chakrabarti et al., 2010; Lawson et al., 2010; Chartoff and Mavrikaki, 2015; Abraham A. D. et al., 2018). Similarly, the efficiency of the long-lasting KOR antagonist norBNI is sex-dependent, probably because of a process induced by oestrogen regulation of G-protein signalling (Reichard et al., 2020). Indeed, estradiol is able to modify the G-coupled-protein action of KOR (Figure 1), decreasing dysphoric effects and enhancing analgesia (Abraham A. D. et al., 2018). Furthermore, the DYN/KOR system plays a crucial role on puberty onset and fertility (Navarro et al., 2009).

## Post-Traumatic Stress Disorder and the Dynorphin/Kappa-Opioid Receptor System

PTSD, previously classified as an anxiety disorder (DSM-IV), is now classified in the DSM-V among “trauma- and stressor-related disorders”. PTSD causes significant impairment in daily functioning and it develops after direct or indirect exposure to an acute life-threatening stress. The estimated lifetime prevalence of PTSD is near 9% (Kessler, 1995; Roque, 2015). Traumatic events may include war, physical violence, sexual abuse, accidents, violent crime, epidemic infections or natural disasters. PTSD includes four major clusters of symptoms observed several months, and even years, after the trauma: 1) re-experiencing of the traumatic event through dreams, flashbacks and intrusive, distressing thoughts; 2) avoidance of trauma reminders; 3) numbing of emotions, negative alterations in mood and cognition; and 4) hyperarousal, characterized by difficulties in sleeping and concentrating, irritability, and hypervigilance (Kessler, 1995; Roque, 2015; María-Ríos and Morrow, 2020). The criteria to meet PTSD (ICD 10) include key symptoms that have to last 6 months: flashback, avoidance of circumstances resembling or associated with the stressor; and inability to recall some important aspects of the trauma, or persistent symptoms of increased psychological sensitivity and arousal. Behavioural PTSD treatments may consist in inducing extinction of the traumatic memory. Indeed, patients suffering from PTSD exhibit deficient extinction recall along with dysfunctional activation of the fear extinction network (Maren et al., 2013; Wicking et al., 2016).

In some studies, PTSD is twice as common in adult women compared to men (Dell’Osso et al., 2011; María-Ríos and Morrow, 2020). This gender difference is already present in adolescence, with girls having more than three times the odds of having the disorder compared to boys (McLaughlin et al., 2013) and may be related to differences in socialization or trauma exposure. Indeed, traumas most commonly associated with PTSD are combat exposure and witnessing violence among men and rape and sexual molestation among women (Kessler, 1995). Biological sex may also impact PTSD development (Garza and Jovanovic, 2017). For example, compared to men, women show a greater reactivity to negative emotional stimuli in key brain structures of the fear circuit (Stevens and Hamann, 2012). We will next briefly review this fear circuitry to then explore its modulation by the DYN/KOR system.

### Neurocircuitry Involved in Stress and Post-Traumatic Stress Disorder

Among the most central brain structures of the fear circuit, there is the hippocampus which is involved in contextual aspects of fear (the environment) and fear generalization (a PTSD symptom). The amygdala is involved in both contextual- and cue-related fear. The lateral (LA) and basolateral (BLA) nuclei of the amygdala project together to the central nucleus of the amygdala (CeA) (Johansen et al., 2011; Izquierdo et al., 2016). The CeA stimulates the hypothalamus and the periaqueductal gray (PAG) to induce three main fear responses (Deng et al., 2016; Lanius et al., 2018): 1) the autonomic response initiated by the lateral hypothalamus involving sympathetic activation (tachycardia, increased blood pressure, change in body temperature, sweating…) preparing the body for physical reactions to danger; 2) the behavioural “fight or flight” defensive response initiated by the dorsolateral and the ventral PAG (active defence behaviour, freezing immobility, running, jumping, aggression); 3) the hormonal stress response, initiated by the paraventricular nucleus (PVN) of the hypothalamus.

The PVN projects to the anterior pituitary allowing the release of the corticotropin-releasing factor (CRF). The anterior pituitary, induces the adreno-cortico-trophic hormone (ACTH) secretion that leads to glucocorticoids release (corticosterone in rodents or cortisol in primates) from the adrenal cortex. Glucocorticoids receptors within the pituitary, the hippocampus and the frontal cortex mediate the negative feedback on hormone release from HPA axis. The ventromedial prefrontal cortex (vmPFC) is also well located to regulate fear learning and memory, as both the prelimbic (PL) and infralimbic (IL) cortices receive extensive projections from the hippocampus and the BLA, and send projections back to the BLA. It is believed that PL and IL play opposing roles in the realm of fear learning: the PL is purported to be necessary for the expression of fear learning, while the IL is thought to be necessary for extinction learning (Izquierdo et al., 2016). A last structure worth mentioning is the bed nucleus of the stria terminalis (BNST) which is more involved in anxiety-like behaviours than conditioned fear. It is extensively connected to the PVN, and would be triggered more by distant or unpredictable threats, compared to the amygdala which is more about proximal and imminent dangers (Avery et al., 2016).

PTSD is characterized by an exaggerated fear and a deficit in fear memory extinction, which may be caused by a PFC-amygdala dysfunction. Most of this mechanism has been documented in rodent models (Izquierdo et al., 2016), but recent clinical findings corroborate them (Åhs et al., 2015; Raij et al., 2018; Yoshiike et al., 2018; Dunsmoor et al., 2019). Interestingly, it has been shown that transcranial magnetic stimulation of the human homologue of IL region of vmPFC enhances fear memory extinction (Raij et al., 2018). PTSD can reprogram fear circuitry in adults, in adolescents and in pediatric PTSD. Several changes in brain structures have been reported, such as a smaller cerebral gray matter volume (Milani et al., 2017), smaller PFC areas (Keding and Herringa, 2015) and larger amygdala (Weems et al., 2015). Furthermore, a prospective study in adolescents suggests that over-activity within a fear network, such as within the amygdala, may increase lifetime vulnerability to develop PTSD after a trauma (McLaughlin et al., 2014).

### Role of Dynorphin/Kappa-Opioid Receptor System in Stress-Related Disorders

**FIGURE 2:**
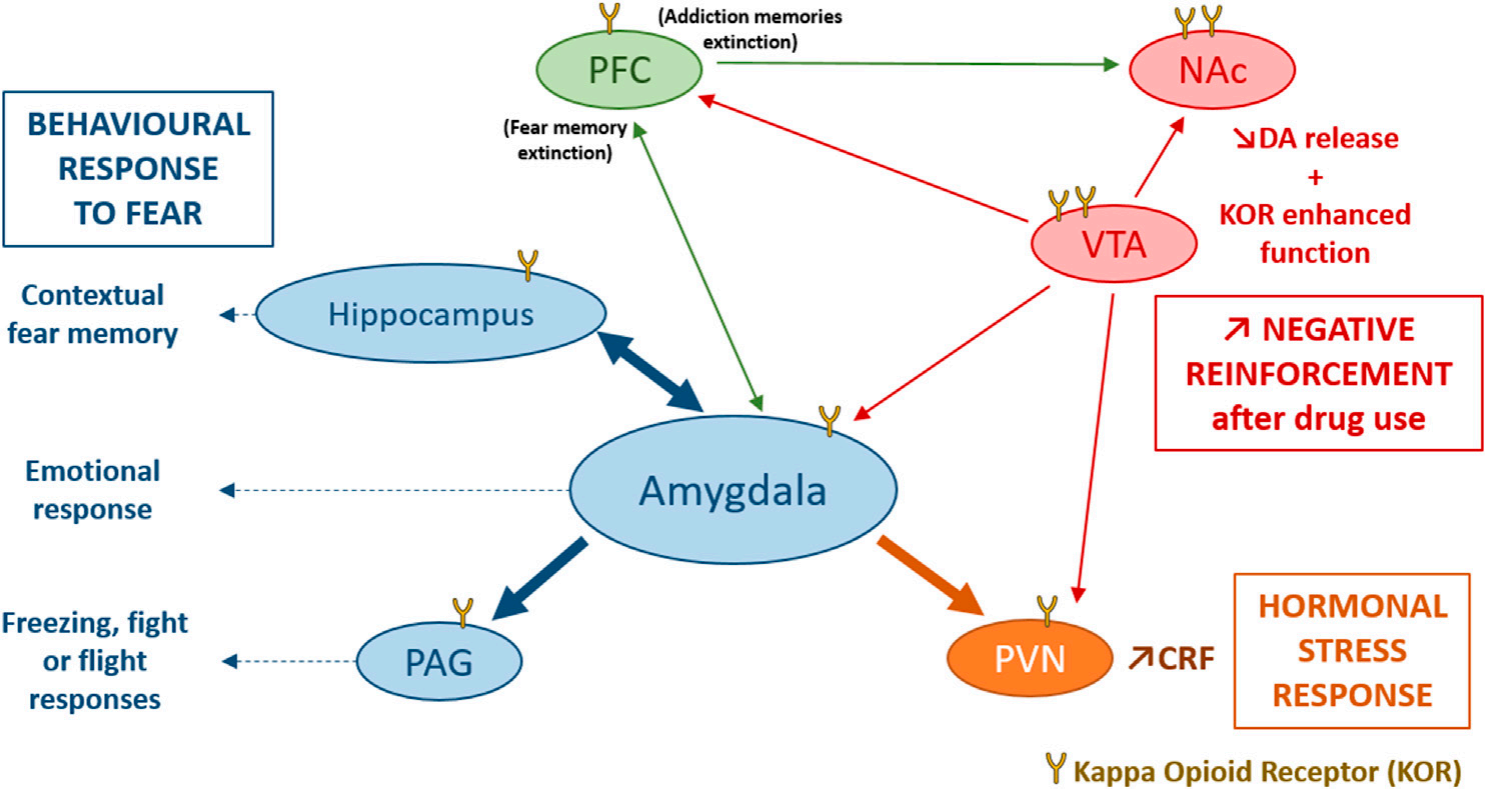
Involvement of the DYN/KOR system in response to stress. KOR activation induce an activation of the hypothalamo-pituitary-adrenal axis mainly through an increase in CRF release by the PVN, participating in the hormonal stress response (Van’t Veer and Carlezon, 2013). The amygdala, the hippocampus and the PAG are directly modulated by KOR and modulate contextual fear memory, emotional response and the freezing, fight or flight responses to fear. Stress could also induce dynorphin release and activates KOR in VTA dopaminergic neurons, inducing a decrease of DA release in several brain structures such as, the PFC, the NAc, the amygdala and the PVN (Karkhanis et al., 2017). After stress or trauma, some interesting processes could be observed: an enhanced function of KOR in VTA, and NAc, an endogenous opioid withdrawal, or an enhanced negative reinforcement after drug use, that may potentiate the risk of comorbid substance use disorder. CRF, Corticotropin Releasing Factor; DA, dopamine; KOR, Kappa Opioid Receptor; NAc, Nucleus Accumbens; PAG, periaqueductal gray; PFC, PreFrontal Cortex; PVN, ParaVentricular Nucleus of the hypothalamus; VTA, Ventral Tegmental Area.

The opioid systems play important roles in regulating the HPA axis (Figure 2). Although the β-endorphin/MOR system contributes to decreasing the HPA axis activation after an acute stress, the DYN/KOR system activates the HPA axis (Bali et al., 2015). KOR are widely expressed in the central nervous system, notably in structures reviewed above, and in the HPA axis that modulate glucocorticoids release (Van’t Veer and Carlezon, 2013; Lanius et al., 2018). Administration of a KOR agonist is able to induce an increase in corticosterone release in rodents (Fuller and Leander, 1984), and of cortisol release in humans (Ur et al., 1997). Among synthetic opioid agonists, those targeting KOR are able to stimulate cortisol release activity after acute administration in primates (Pascoe et al., 2008) and in humans (Ur et al., 1997). Inversely, the long-acting KOR antagonist nor-BNI (Allen et al., 2013), or the short-acting KOR antagonist LY2444296 (Valenza et al., 2017), both reduce corticosterone release following diverse stressors, chronic cocaine administration (Valenza et al., 2017) or food restriction (Allen et al., 2013).

The most widely investigated interactions of KOR with the HPA axis are those with CRF (Koob et al., 2014; Crowley and Kash, 2015) a neuropeptide that can also mimic physiological and behavioural response of stress when administered exogenously (Hauger et al., 2009; Van’t Veer and Carlezon, 2013; Koob et al., 2014). In the CeA, DYN containing neurons have been demonstrated to occasionally co-express CRF (Marchant et al., 2007). DYN and CRF are also co-expressed in the PVN of the hypothalamus (Roth et al., 1983), and the hypothalamic supra-optic nucleus (Meister et al., 1990). In addition to this anatomical overlay between the two peptidergic systems, it has been shown that the DYN/KOR system is able to influence CRF expression in the PVN (Wittmann et al., 2009) and in the CeA (Wittmann et al., 2009; Negrete et al., 2017), while CRF mRNA was found decreased in PDYN-KO mice. Inversely, CRF is able to induce DYN release in the rat striatum (Sirinathsinghji et al., 1989) and the mouse spinal cord (Song and Takemori, 1992). Beside the release of stress hormones, the CeA exerts its function on the flight reactions via the dorsolateral PAG which is directly modulated by KOR. Systemic administration of Nor-BNI reduces the flight reaction induced by PAG stimulation. Furthermore, microinjection of Nor-BNI into the PAG causes the same panicolytic-like effect (Maraschin et al., 2017). On the other hand, anxiety behaviour can also result from a KOR modulation of the BLA to BNST input (Crowley et al., 2016).

The data about the effects of stress and active defense behaviours in relation to the DYN/KOR system (Figure 2) in rodents can be found in studies that used models of anxiety- and depressive-like behaviours (anhedonia, deficient grooming, learned helplessness…). Several studies have shown that KOR antagonists can prevent the behavioural consequences of stress (Van’t Veer and Carlezon, 2013). Thus, after diverse stressors (*e.g.*, CRF administration or withdrawal following repeated administration of drugs of abuse) these antagonists are able to induce anxiolytic-like effect in the elevated plus maze (EPM) (Bruchas et al., 2009; Jackson et al., 2010; Valdez and Harshberger, 2012) and the open field (OF) tests (Wittmann et al., 2009), and decrease immobility time in the forced swim test (FST) used to screen antidepressant drugs (Newton et al., 2002; McLaughlin et al., 2003; Shirayama et al., 2004). Tonic activation of KOR by DYN does not appear to play a role in baseline anxiety- or depression-like behaviours. Indeed, constitutive global PDYN-KO and KOR-KO mice have similar performances in the EPM and in the sucrose preference test compared to wild-type animals (Negrete et al., 2017). However, using conditional KOR-KO mice, a deletion of KOR in the amygdala results in an increased anxiety-like behaviour (Crowley et al., 2016).

Several lines of evidence suggest sex differences in the modulation of depression-related behaviour by DYN/KOR (Chartoff and Mavrikaki, 2015). It was observed that KOR activation is less dysphoric in female than male rodents (Russell et al., 2014; Abraham A. D. et al., 2018), but this effect seems to be highly dependent on the KOR agonist dose used (Robles et al., 2014). Interestingly, the KOR antagonist norBNI decreases immobility in the FST in male mice, without modification in females (Laman-Maharg et al., 2018), while in an intracranial self-stimulation model, the depressive-like effect of the KOR agonist U50,488 is weaker in female rats compared to males, independently of gonadal hormones (Russell et al., 2014).

Concerning conditioned fear memory, decrease expression of cue-dependent fear and fear-potentiated startle were observed after a KOR antagonist administration, while it facilitated extinction of fear in a context-dependent manner (Fanselow et al., 1991; Knoll et al., 2007; Cole et al., 2011). In contrast, in another study, mice lacking DYN or mice treated with a KOR antagonist displayed the opposite effect, an increased contextual fear and a delayed fear extinction (Bilkei-Gorzo et al., 2012). Furthermore, the role of KOR in fear conditioning depends on the brain area. In the NAc, KOR may downregulate attention to conditioned stimuli that are redundant or non-informative predictors of shocks (Iordanova et al., 2006). A transient activation of KOR in the CA3 region of the hippocampus impairs both the acquisition and the consolidation of contextual fear-related memory (Daumas et al., 2007), while KOR antagonism in either the BLA or CeA decreased conditioned fear in the fear-potentiated startle paradigm (Knoll et al., 2011). Furthermore, an effective extinction of the fear potentiated startle is associated with a 67% reduction in KOR mRNA in the BLA (Knoll et al., 2011). Finally, knockdown of DYN or CRF signalling in CRF-expressing CeA neurons decrease the expression of both contextual- and cued-induced conditioned freezing (Pomrenze et al., 2019).

Therefore, overall, considering data obtained from animal models of anxiety-, depression-like behaviours, it appears that activation of the DYN/KOR system is associated with more anxiety or dysphoria. Although the DYN/KOR system is clearly implicated in fear memory expression there are contrasting findings regarding its exact role.

## Substance Use Disorder and the Dynorphin/Kappa-Opioid Receptor System

Recreational use, or initiation of psychotropic substance use, generally results in a hedonic state: a pleasant emotional response, called positive reinforcement (Fields and Margolis, 2015). This reinforcement, which involves activation of the reward system, could lead to SUD, although this depends on an individual’s vulnerability. To define drug addiction, the DSM-V employs the terminology “Substance use disorders” (SUD) that takes into account the “harmful use” (abuse) and the “out-of-control use” (dependence) (Norko and Fitch, 2014). SUD are frequently comorbid with diverse neurologic and psychiatric disorders, including anxiety, depression (Gómez-Coronado et al., 2018), schizophrenia (Hunt et al., 2018), borderline personality (Lee et al., 2015), attention deficit/hyperactivity (Katzman et al., 2017) and PTSD (Jacobsen et al., 2001). Interestingly, in all the psychiatric pathologies associated with SUD, there is evidence for involvement of the HPA axis activation (CRF release), certain neurotransmitter systems (DA, 5-HT, GABA, and glutamate) and mediators of inflammation (Gómez-Coronado et al., 2018).

In drug addiction, depending on types and stages of SUD, there are at least two opposite adaptive processes: the first one is associated with DA release, while the second one is associated with suppression of DA release. Indeed, the DA release in the brain associated with pleasure*,* as a consequence of drug administration or its anticipation, causes an altered excitatory/inhibitory balance. To compensate this imbalance, after drug metabolization, the suppression of DA release is observed, which can induce a negative affective state. In a subject developing addiction, by escalating drug-taking behaviour, the first process (hedonic state) is less and less pronounced, while the second process progressively increases. The later induces anhedonia and dysphoria, called negative reinforcement. During the dependence process, the drug-seeking behaviour for the substance is, from the motivation point of view, much more due to the avoidance of negative reinforcement than the initial positive reinforcement (Karkhanis et al., 2017). This negative reinforcement involves, among others, the DYN/KOR system that has been found to be up-regulated during addiction (Walker and Koob, 2008).

### Neurocircuitry Involved in Reward and Addiction

The VTA is the key brain structure for mediating the rewarding effects of a drug through the activation of mesocorticolimbic DA pathways, while NAc, amygdala, PFC and BNST are major targets of the VTA dopaminergic neurons (Swanson, 1982). All drugs of abuse are known to increase DA release in the NAc. This DA increase is obtained either by inhibition of VTA GABAergic interneuron projecting to dopaminergic neurons (observed with MOR agonists and cannabinoids), or by inhibiting DA reuptake in the NAc through a direct blockade of the DA transporter (DAT; observed with cocaine). Diverse brain areas could be involved in SUD, depending of the stage or drug used. For example, the dorso-medial and lateral striatum, the BLA and CeA are involved in developed and established complulsive habits (Lüscher et al., 2020). The locus coeruleus is involved in withdrawal and relapse in chronic use of psychostimulants and alcohol (España et al., 2016), but also in withdrawal from morphine with a pDYN enhanced expression (McClung et al., 2005). serotoninergic neurons of the raphe nuclei, most known for their involvement in mood and dysphoria, also play an important role in addiction most likely in relation with impulsivity regulation (Kirby et al., 2011).

Circuits of the PFC, involved in fear behaviours, overlap the circuit that regulates extinction of conditioned responses associated with drug intake. Projections of the vmPFC regulate, *via* glutamate, locomotor drug sensitization, drug-seeking and drug withdrawal, similarly to fear extinction. On one hand, the IL cortex not only decreases fear via the CeA, but also sends excitatory projections to the NAc shell, which indirectly inhibit motor responses involved in drug seeking. On the other hand, the prelimbic (PL) cortex, that is rather excitatory in conditioned fear, also favours drug seeking. Therefore, IL favours extinction of addiction, as of fear, and PL favours expression of acquired addiction behaviours, as conditioned fear (Peters et al., 2009). Another structure involved in reward as much as anxiety is the BNST. The latter would be involved in the negative affective state generated by drug withdrawal. Together with the NAc and the amygdala, the BNST would be involved in the generation of dysphoria triggering relapse after a long period of abstinence (Avery et al., 2016).

### The Dynorphin/Kappa-Opioid Receptor System During Addiction

The mesolimbic and mesocortical DA pathways are central in the effects of DYN/KOR system in the brain (Van’t Veer and Carlezon, 2013; Tejeda and Bonci, 2019). In the mesolimbic structures, KOR activation (notably by stress or SUD) decreases DA release in the BLA, and causes a direct inhibition of DA neurons firing in the NAc (Karkhanis et al., 2017). In the VTA, the DYN inhibits DA neurons in two ways: a direct negative feedback loop following D1 receptor activation, and indirectly through inactivation of cholinergic interneurons (Karkhanis et al., 2017). It was initially postulated that activation of KOR in the VTA induces a decreased of DA release (Dalman and O’Malley, 1999) and glutamate release (Margolis et al., 2005) that may produce the negative reinforcement effects of KOR agonists (Bals-Kubik et al., 1993). However, a direct activation of KOR in NAc, PFC or lateral hypothalamus, could also mediate such effects (Bals-Kubik et al., 1993; Al-Hasani et al., 2015). Activation of KOR located on dopaminergic terminals in the PFC produces a local reduction of DA release and is sufficient by itself to prevent the conditioned place aversion produced by systemic U69,593, a KOR agonist (Tejeda et al., 2013).

Several studies suggest that KOR activation in dopaminergic VTA neurons may disrupt behavioural inhibition (Abraham AD. et al., 2018). KOR may even inhibit fear memory acquisition in the hippocampus (Daumas et al., 2007), while generating anxiety-like responses in the amygdala (Knoll et al., 2011). In the NAc, while activation of KOR in the ventral part leads to aversion, an opposite behaviour is reported following KOR activation in the dorsal part of NAc that drives preference/reward behaviours (Al-Hasani et al., 2015). Furthermore, the DYN/KOR system reduces the development of addiction, but may also potentiate reinstatement after extinction (Van’t Veer and Carlezon, 2013; Karkhanis et al., 2017). The complex and large distribution of KOR in the VTA and the NAC could explain such discrepancy. Furthermore, KOR activation in the VTA inhibits both GABA and DA neurons projecting on the PFC, the NAc and the BLA (Van’t Veer and Carlezon, 2013; Karkhanis et al., 2017).

KOR do not act exclusively on DA neurons to modulate aversion/dysphoria in the potentiation of drug reward. The stress-induced DYN release activates KOR in serotonergic neurons and contributes to reinstate drug seeking (Land et al., 2009). In a microdialysis study, a KOR agonist decrease local serotonin (5-HT) efflux when infused into the dorsal or median raphe as well as in the NAc (Tao and Auerbach, 2002). The KOR-mediated modulation of mood and drug reward probably involves activation of 5-HT1B receptors in the NAC (Fontaine et al., 2022) and the antidepressant-like effect of KOR antagonist could somehow be related to decrease function/density of the serotonin reuptake transporter, the primary target of SSRIs (Sundaramurthy et al., 2017).

### Effects of Kappa-Opioid Receptor Ligand Treatments

A first approach concerning the use of the DYN/KOR system as a therapeutical target in addiction is to administer agonists during the acquisition of drug dependence. In preclinical studies, the conditioned place preference (CPP) test evaluates the drug reward effect, by associating the drug administration with a specific environment (Prus et al., 2009). In this model, KOR agonists would exert a dysphoric effect as they induce place aversion (Van’t Veer and Carlezon, 2013; Cahill et al., 2014; Margolis and Karkhanis, 2019); *i.e.,* rodents actively avoid a context previously associated with a KOR agonist. However, optogenetic studies show that dynorphinergic cell stimulation creates either aversive (anti-reward) effects when stimulating the ventral NAc shell, or reward effects when stimulating the dorsal NAc shell (Al-Hasani et al., 2015). Furthermore, there are gender effects in this KOR-induced dysphoria: with a low dose of KOR agonist, female, but not male, mice developed a place aversion, while with a high dose, male but not female mice developed this aversion (Robles et al., 2014). Interestingly, if a social defeat stress is induced before KOR agonist administration, it inhibits the aversive effect of a low dose of KOR agonist in females, without modification of the effects induced by a high dose of KOR agonist in male mice (Laman-Maharg et al., 2017), suggesting that prior stress modifies the dysphoric effect of KOR in females.

Morphine and alcohol are also used to induce CPP, and this behaviour is blocked by a pre- or co-treatment with U-50,488H or E-2078, another KOR agonist (Funada et al., 1993; Matsuzawa et al., 1999). The blockade of morphine-induced CPP by KOR agonists may result from a reduction of DA release in the NAc (Funada et al., 1993), since DYN is able to decrease basal and cocaine-induced rise in striatal DA levels (Zhang et al., 2004). Similarly, U50,488H administration during alcohol conditioning inhibits both alcohol-seeking behaviour and alcohol-induced locomotor activation (Logrip et al., 2009). An increase of alcohol self-administration was observed following KOR antagonist treatment in rats during the acquisition phase of a self-administration behaviour, while KOR agonist administration was able to reduce self-administration. This effect could be due to a direct modulation of the reward circuitry (Mitchell et al., 2005). In the 1990’s, it was proposed that KOR agonists could be used to prevent the initiation of behavioural sensitization and alterations in mesolimbic DA neurotransmission (Shippenberg and Rea, 1997). However, such DYN effect on early exposure to drug remain unexploited clinically as KOR agonists would need to be co-administered with the addictive drug. Inversely, it has been proposed that stress induced DYN release could produce a dysphoric state that increase the rewarding valence of addictive drugs (McLaughlin et al., 2003, 2006), consequently KOR agonists may thus potentiate addiction development.

The DYN/KOR activation is essential after an early consumption of drugs to equilibrate brain DA and limit addictive properties of abuse drugs. In a rat model of heroin self-administration, DYN expression in the striatum was enhanced during withdrawal periods but not during acute administrations (Cappendijk et al., 1999). In another study, it has been shown that DYN expression is upregulated 3–24 h after methamphetamine administration in the dorsal striatum. This effect is associated with dopaminergic toxicity that creates oxidative burdens, microgliosis, and pro-apoptotic changes (Dang et al., 2018). DYN basal expression can also predict the vulnerability to develop an addictive behaviour. Comparing two strains of rats, Nylander and colleagues (1995a) have shown that Lewis rats, that have a higher propensity to self-administer various drugs of abuse than Fischer rats, display lower basal DYN levels in the *substantia nigra*, striatum, VTA and the pituitary gland. Moreover, chronic morphine treatment and opiate withdrawal induced different regulations of DYN and enkephalin in the two strains (Nylander et al., 1995a; 1995b). Other preclinical studies exploiting inter-strain differences in PDYN genes expression in the NAc, suggest that a high expression of PDYN may protect against morphine addiction by limiting drug-induced reward (Gieryk et al., 2010). In addition, it has been shown that after cocaine withdrawal DYN’s action on GABAergic and glutamatergic neurons is altered in the ventral palidus, a structure involved in relapse behaviour (Inbar et al., 2020).

Although KOR agonists could somehow inhibit the positive reinforcement process during a SUD development (Shippenberg and Rea, 1997), once dependence and tolerance are established, the best therapeutic strategy remains KOR antagonists that reduce the relapse related to withdrawal-induced anxiety. KOR antagonists are able to alleviates alcohol withdrawal-induced anxiety and reduce alcohol self-administration in rats (Schank et al., 2012), confirming role of KOR in stress-induced relapse after withdrawal. In other words, the DYN/KOR system is involved in stress-induced vulnerability, not only during addiction development, but also during the phase of relapse-risk after drug withdrawal (Karkhanis et al., 2017).

### Traumatic Stress Induces Vulnerability to Addiction

As already mentioned in the Introduction, there is a high prevalence of SUD/PTSD comorbidity. As in a vicious circle, the co-occurrence of PTSD and SUD makes an individual symptoms more severe and more difficult to treat (María-Ríos and Morrow, 2020). In particular, among SUD patients, the risk of relapse is strongly enhanced in case of comorbid PTSD (Norman et al., 2007). Furthermore, during a traumatic event, both endorphin and DYN levels increase in the brain during the so-called fight or flight responses (Kavushansky et al., 2013; Lanius et al., 2018). However, after a trauma, whereas KOR function may be enhanced in some brain areas such as the BNST, the NAc, the VTA, in other limbic structures MOR density is reduced, producing a period of endogenous opioid withdrawal (Lanius et al., 2018). PTSD patients may counteract these negative effects by using drugs of abuse (Volpicelli et al., 1999).

The choice of the drug of abuse selected by PTSD patients could determine the severity and the nature of PTSD symptoms. The PTSD-related symptoms clusters could be used in order to distinguish potential mechanisms underlying those PTSDs comorbid with SUD (Dworkin et al., 2018). Among the three symptoms related to PTSD, alcohol use is only associated with avoidance symptoms (Lane et al., 2019). Cocaine is associated with hyperarousal symptoms and sedative/hypnotic/anxiolytic use is associated with numbing of emotions (Dworkin et al., 2018). Interestingly, opiates, generally MOR agonists, use is particularly important in comorbid PTSD and SUD (Danovitch, 2016; Elman and Borsook, 2019), this may result from the fact that avoidance symptoms (Phifer et al., 2011), numbing of emotions (Dworkin et al., 2018), and hyperarousal symptoms are strongly associated with opiates use and misuse (Dworkin et al., 2018; Takemoto et al., 2020).

**FIGURE 3:**
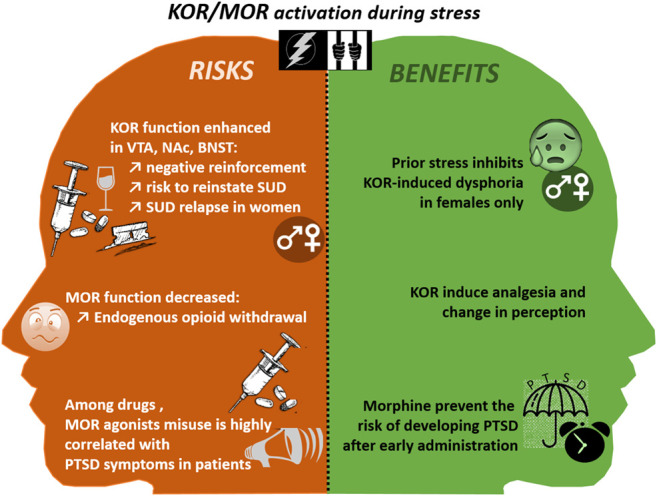
The risks and benefits of MOR and KOR activation during and after traumatic events. BNST, Bed Nucleus of the stria terminalis; KOR, Kappa Opioid Receptor; NAc, Nucleus Accumbens; PTSD, Post Traumatic Stress Disorder; SUD, Substance Use Disorder; VTA, Ventral Tegmental Area.

Both current and past PTSD periods resulting from non-combat-related exposures are strong risk factors for opiates (MOR agonists) use and misuse (Takemoto et al., 2020). Inversely, among patients treated for heroin dependence, the prevalence of PTSD has been estimated to 66% (Mills et al., 2018), suggesting again that self-medication could play a role in this opioid-use/PTSD comorbidity. Indeed, it is now well documented in the literature that PTSD is associated with diverse pain disorders, in civilian patients without injuries (Schwartz et al., 2006; Phifer et al., 2011) or with a traumatic brain injury (Bryant et al., 1999), and in war veterans (Wagner et al., 2000). As illustrated in Figure 3, morphine administered from 1 to 48 h after trauma, inhibits the trauma-related memory consolidation (Roque, 2015), thus preventing PTSD symptoms, in children (Saxe et al., 2001) and adults (Schönenberg et al., 2005), notably by reducing separation-anxiety in children (Saxe, 2006), in a dose-dependent manner (Bryant et al., 2009).

Opioids can also produce anti-stress effects, notably through the modulation of both endorphin/MOR and DYN/KOR systems (Schmidt et al., 2014; Bali et al., 2015). In preclinical studies, it has been shown that DYN/KOR activation through a chronic stress (e.g., repeated forced swim test), or through selective agonist administration significantly potentiate the magnitude of nicotine CPP acquisition (Smith et al., 2012). Similarly, KOR activation induced by repeated forced-swim stress prior to cocaine CPP is both necessary and sufficient for potentiation of cocaine’s reinforcing actions (Schindler et al., 2010). Independently of associative learning mechanisms, stress may directly enhance the rewarding value of cocaine by a DYN-dependent mechanism in the amygdala (Schindler et al., 2010).

The DYN/KOR system activation could also reinstate a reward memory after extinction of cocaine-induced CPP, suggesting that KOR is involved in stress-induced vulnerability to addiction by acting directly on stress-induced neuroplasticity (Land et al., 2009). Traumatic stress (e.g., brief cold-water swim), leads to a persistent constitutive activation of KOR and abolish the long-term potentiation of GABAergic synapses in the VTA, leading to a reinstatement of cocaine seeking in a rat self-administration model after behavioural extinction (Polter et al., 2017). Furthermore, a long-lasting KOR antagonist, suppresses stress-induced, but not cocaine-recall, reinstatement of cocaine CPP (Carey et al., 2007). Moreover, a cocaine CPP is reinstated after either a KOR agonist administration or a chronic social defeat (Bruchas et al., 2011; Smith et al., 2012). Stress-induced cocaine CPP reinstatement is inhibited by the antagonist nor-BNI (Bruchas et al., 2011; Smith et al., 2012), and is absent in knockout mice lacking the KOR or the PDYN genes (McLaughlin et al., 2003; Redila and Chavkin, 2008; Land et al., 2009). Similarly, nor-BNI has been shown to inhibit stress-induced reinstatement of nicotine-CPP after foot-shocks (Nygard et al., 2016) or ethanol-CPP after forced swim (Sperling et al., 2010). This stress-induced DYN/KOR system activation, involved in drug-seeking behaviour, has been shown to be dependent on various mechanisms, including a transient rise in 5-HT transport in the ventral striatum (Bruchas et al., 2011; Smith et al., 2012), the activation of KORs located in serotonergic neurons of the dorsal raphe nucleus (Land et al., 2009), a KOR mediated Gαi signalling pathway within BLA neurons (Nygard et al., 2016) or an increased noradrenergic neurotransmission in the locus coeruleus (Al-Hasani et al., 2013). Besides, alcohol-dependent rodents with an extended period of abstinence submitted to either a stress (a 20 min immobilization) or injection of the agonist U50,488, develop an anxiogenic-like behaviour with a decrease time spent in the open arms in the EPM. In this model of enhanced responsiveness to stress, nor-BNI administration inhibits this increase in anxiety (Gillett et al., 2013).

Finally, again, gender differences could be observed concerning the PTSD-induced SUD vulnerability, during the drug dependence acquisition, withdrawal or relapse phases. Women’s risk of addiction increase more rapidly than men from the initial use to addiction, and this is true for all drugs of abuse (Brady and Randall, 1999). Trauma history and current trauma-related symptoms are significantly associated with relapse in women, but not men, in chronic and binge alcohol use (Heffner et al., 2011) or cocaine (Hyman et al., 2008). It was argued that being a woman and being a previous user of cocaine or opiates were the strongest predictors of PTSD (Saladin et al., 1995). Similarly, the use of illicit drugs is strongly associated with both sexual and physical assault in women (Kilpatrick et al., 1997). Childhood victimization is higher in alcoholic women compared with non-alcoholic ones (Miller et al., 1993). There are also effects of experience and culture on vulnerability to addiction that can differentially affect males and females (Becker, 2016). At the preclinical level, the sexually dimorphic balance of drug-induced DA release in the dorsolateral striatum and the NAc, could also explain the gender difference (Becker, 2016). The latter may also result from sexually dimorphic: 1) vulnerability to opiate addiction dependent from polymorphisms in the PDYN gene (Clarke et al., 2012), 2) MOR/KOR heterodimerization (Chakrabarti et al., 2010), or 3) gonadal hormone modulation by the DYN/KOR system (Eghlidi et al., 2010; Lawson et al., 2010). Although recently explored in addiction alone (Mitchell et al., 2005), stress (Robles et al., 2014; Russell et al., 2014; Laman-Maharg et al., 2017, 2018; Abraham A. D. et al., 2018; Williams and Trainor, 2018) or pain (Chartoff and Mavrikaki, 2015; Abraham A. D. et al., 2018), there is no experimental research on the sexually dimorphic effect of DYN/KOR system in addiction vulnerability triggered by stress. Such information shall be necessary to explain the great prevalence of SUD/PTSD comorbid situations in women.

## Conclusion

As far as PTSD and addiction are concerned, several lines of evidence indicate that when these disorders are comorbid, their symptoms are more severe and treatment more difficult than with either disease alone (Taylor et al., 2017). One strategy put forward in this review is to use a KOR ligand to treat this comorbidity. For example, we could envision treating the dysphoric state associated with drug withdrawal with an antagonist (Carroll and Carlezon, 2013; Banks, 2020) in conjunction with an exposure therapy to decrease the incidence of PTSD symptoms. Indeed, the activation of DYN/KOR system is crucial during stress responses, and either traumatic stress or addiction development increases its function (Van’t Veer and Carlezon, 2013; Karkhanis et al., 2017), leading to increased risk of SUD in patients that lived traumatic events. Alternatively, a KOR biased-agonist, averting the β-arrestin 2-related signalling mediating dysphoria would be advantageous compared to MOR agonists for pain management and/or prevention of PTSD development, as such agonist could prevent dependency (Spetea and Schmidhammer, 2022). Nevertheless, future pharmacological studies should better explore gender effects for these KOR ligands, since agonists have good analgesics properties but inconsistent dysphoric side effects in women (Chartoff and Mavrikaki, 2015).

To better understand the addiction to opiates it is essential to take into consideration the wide distribution of KOR into the fear/stress circuitry, including the amygdala, the PAG, the frontal cortex, the hippocampus, the BNST and the HPA axis. KOR activate the HPA axis (Van’t Veer and Carlezon, 2013) and DYN is often co-expressed with CRF in regions such as the CeA and the PVN (Crowley and Kash, 2015). In view of these observations, the therapeutic-like action of KOR antagonists, in animal models of anxiety as well as in models commonly used to screen antidepressant drugs, is not surprising (Spetea and Schmidhammer, 2022). Furthermore, although controversial at this point, KOR antagonists may effectively reduce associative fear memories, by acting on some structures involved in traumatic fear memory formation (Daumas et al., 2007; Cole et al., 2011; Knoll et al., 2011). Here again, there seems to be important gender effects in these models.

In the so-called opponent process theory of addiction (Koob et al., 1989), the DYN/KOR system appears as a crucial element explaining decrease dopaminergic transmission associated with dysphoria, contrasting with the initial hedonic hyperdopaminergic state (Karkhanis et al., 2017). In addition, brain areas involved in stress, along the HPA axis and the fear circuit, overlap with those involved in addiction. In particular, the VTA innervated BNST and NAc regulated by the same vmPFC PL and IL areas as is the amygdala to regulate extinction. KOR activation and the hypodopaminergic state associated with aversion thus involve as much the amygdala as the NAc (Van’t Veer and Carlezon, 2013). Trauma induces a KOR function increase in the VTA, the NAc and the BNST, that leads to endogenous opioid withdrawal and enhanced negative reinforcement after drug consumption. In addition, KOR dysphoric effect is dose, stress and gender dependent. Despite these contradictions, we believe that the best pharmacological strategy remains the development of KOR antagonist to reduce relapse, withdrawal-induced anxiety and PTSD predisposition.

Acute morphine treatments, can effectively reduce pain in patients suffering from traumatic stress, and they are initially beneficial whether they are associated with traumatic injury or not (Saxe et al., 2001; Schönenberg et al., 2005). Surprisingly, a short-term administration, right after a traumatic event, decreases the risk to develop PTSD, probably by reducing the trauma-related memory consolidation (Saxe, 2006). However, on the other hand, among all the drugs of abuse, opiates and other MOR agonists, in particular heroin, most commonly trigger the SUD-PTSD comorbidity, and more obviously in women (Najavits et al., 1997). The fact that the DYN/KOR system is directly interacting with the stress axis, the fear and the reward circuitries may explain this intriguing observation.

### General Conclusion

With advances in genetics, molecular biology and neurobiology, our understanding of the central role of the DYN/KOR system in the mechanisms of addiction and traumatic stress progresses. The activation of DYN/KOR system now appears crucial in stress responses. Traumatic stress like addiction increase the function of this system. Most research has been made so far in male preclinical models, and thus more research on female models is crucially needed, in view of the gender-dependent differences in DYN/KOR system and the great prevalence of SUD/PTSD in women. Therapeutic strategies, targeting the inactivation of KOR are very promising not only for the treatment of SUD or PTSD alone, but also for the SUD/PTSD comorbidity.
